# 

**Published:** 1918-07

**Authors:** 


### ANNOUNCEMENTS

---

### National Societies

National Dental Association, Chicago, August 5, 6, 7, 8 and 9.

National Association of Dental Faculties, Chicago, Congress Hotel, Green Room, August 2, 3, 1918.

National Association of Dental Examiners, Chicago, 1918.

Xi Psi Phi Fraternity, Chicago, August 5, 1918.

Delta Sigma Delta Fraternity, August 5, 1918, Chicago, Congress Hotel.

Psi Omega Fraternity National Alumni Chapter, Chicago, August 5, 1918. Business session, 3 p. m., New Ball Room, Auditorium Hotel. Banquet, 7 p. m., Florentine Banquet Hall, Congress Hotel.

Association of Military Dental Surgeons, Chicago, August 7, 1918.

American Society of Orthodontists and American Academy of Periodontia, Edgewater Beach Hotel, Chicago, August 1-3, 1918.

---

### National Dental Association

*Sixtieth Anniversary of Organized Dentistry and Dedication of Black Memorial* will be celebrated August 5-9, 1918, by rendering an unusual literary and clinical program at the Auditorium and Congress Hotels, Chicago, Illinois.

*House of Delegates—Monday, August 5.* The Registration office for this great meeting will be open Monday morning at 8:30. The delegates to the House of Delegates will present their credentials and be seated at 10 a.m. This will be the big official business day of the meeting.

*Seminar—The Management of Pulpless Teeth—Tuesday, August 6, 9:30 a.m.* Six of the best known scientific research authorities will submit "evidence and not opinions" on this subject.

*Three Section Meetings—Tuesday, August 6, 2:00 p.m.* The Section on Prosthetic Dentistry and Crown and Bridge Work will render a program on "Occlusion and Variable Occlusion" and "A Scientific Technic in the Construction of an Artificial Denture."

The Section on Oral Surgery and Anesthesia will present a program on "Cleft Palate and Harelip Procedures" and "Recent Progress in the Management of War Injuries of the Face and Jaws."

The Section on Orthodontia and Periodontia have arranged for the following program: "A Classification of Pathological Conditions of the Mouth," "Physiological Factors as Related to the Peridental Membrane, Cementum and Bone in Tooth Movement," "Orthodontic Appliances and Gingival Tissue," "The Histology and Development of Cementum," "The Duty and Responsibilities of the Orthodontist in the Present War," "The Necessity for Orthodontic Interference in Malocclusion of the Teeth."

*Military Dentistry—War Surgery—Tuesday, 8:30 p.m.* The speakers at this session will be surgeons of national and international reputation.

*Unusual Section Programs—Six Sections and Oral Surgery Clinics—Wednesday, August 7, 9:00 a.m.* The Sections on Operative Dentistry, Materia Medica and

Therapeutics—Organization, Mouth Hygiene and Public Service—Research—The Preparedness League of American Dentists and General Oral Surgery Clinics will each present their programs in various rooms in the Auditorium and Congress Hotels.

*2:00 p.m.* The Sections on Prosthetic Dentistry and Crown and Bridge Work—Oral Surgery and Anesthesia and Orthodontia and Periodontia will hold their second session Wednesday, at 2:00 p.m.

*Canadian Dentists, Our Guests—Wednesday, August 7, 8:00 p.m.* How fitting it is, when the young men of the allied countries are freely and willingly giving their lives in the terrible conflict which is absorbing the attention of the entire world, when men and women of every station in life are making their first business the winning of the war, that the dentists of Canada and the United States should unite in holding their national meetings. The Canadian Dental Association will be the guests of the National Dental Association at this meeting and the program for the Third General Session for Wednesday evening will be furnished entirely by this society.

*International General Clinic—150 Clinicians—Thursday, August 8, 9:00 a.m.* Clinics will be given by members of the Canadian Dental Association, representatives of forty states and Alaska. Unit Clinics, Special Clinics on Root Canal and Crown and Bridge Work.

*Luncheon to Our Ex-President.* Former Presidents of the American, Southern and National Association to be the guests of the Association at a luncheon in their honor by the members of the profession, who desire in this small way to show their appreciation of the valuable services they have rendered the profession and also to pay a just tribute to the Founders and Builders of Organized Dentistry.

*Dedication of the Black Memorial Monument—Thursday, August 8, 2:00 p.m.* It has now been almost three years since the dental profession was deeply grieved to hear of the death of Doctor G. V. Black. Those who were present at the Panama-Pacific Dental Congress at San Francisco will never forget, when his death was announced just at the opening of the Congress, how the cloud spread over that vast audience. In matters pertaining to dentistry, Dr. Black was easily the master. If the world misses him, as it surely does, how much more do we miss him? It was our pleasure and privilege to live with him, speak to him and feel free to consult him at any and all times. By his death, the dental profession has lost one of its most cherished members; the world has lost a benefactor. All that was needed to erect a monument to his memory was for some one to say the word. That word has been said, and dentists from nearly every state in the Union, as well as from many foreign countries, have contributed to the fund for the purpose. What could be more appropriate, therefore, than to unveil the monument to this great man when the National Dental Association is holding its annual meeting in the city, where, during the last years of his life, he lived and worked.

*Patriotic Service—Thursday, August 8, 8:00 p. m.* Men of international and national reputation will be on the program for this unusual meeting, men who are the recognized leading statesmen, surgeons and divines of this age. It is quite fitting in this great war crisis that the dental profession should show its patriotic spirit by pledging anew its loyalty and devotion to our country.

*General Session and Section Meetings—Friday, August 9, 9:00 a. m.* The Sections on Operative Dentistry, Materia Medica and Therapeutics, Organization, Mouth Hygiene and Public Service, Research, and the House of Delegates and the last general session.

